# MCH Workforce Capacity: Maximizing Opportunities Afforded by a Changing Public Health System

**DOI:** 10.1007/s10995-018-02728-7

**Published:** 2019-01-22

**Authors:** Ilana G. Raskind, Theresa Chapple-McGruder, Dara D. Mendez, Michael R. Kramer, Karen D. Liller, Dorothy Cilenti, Martha Slay Wingate, Brian C. Castrucci, Elizabeth Gould, Caroline Stampfel

**Affiliations:** 1 Department of Behavioral Sciences & Health Education, Rollins School of Public Health, Emory University, 1518 Clifton Rd. NE, GCR 523, Atlanta, GA 30322 USA; 2 de Beaumont Foundation, 7501 Wisconsin Avenue, Suite 1310E, Bethesda, MD 20814 USA; 3 Department of Epidemiology, Graduate School of Public Health, University of Pittsburgh, 130 Desoto St., PUBHL 5130, Pittsburgh, PA 15261 USA; 4 Department of Epidemiology, Rollins School of Public Health, Emory University, 1518 Clifton Rd. NE, Atlanta, GA 30322 USA; 5 College of Public Health, University of South Florida, 13201 Bruce B. Downs Blvd., MDC 56, Tampa, FL 33612 USA; 6 Department of Maternal and Child Health, Gillings School of Global Public Health, University of North Carolina, Chapel Hill, 402A Rosenau Hall, CB #7445, Chapel Hill, NC 27599 USA; 7 Department of Health Care Organization & Policy, University of Alabama at Birmingham School of Public Health, 1665 University Boulevard, Birmingham, AL 35294 USA; 8 Association of State and Territorial Health Officials (ASTHO), 2231 Crystal Drive, Suite 450, Arlington, VA 22202 USA; 9 Association of Maternal & Child Health Programs (AMCHP), 1825 K Street NW, Suite 250, Washington, DC 20006 USA

**Keywords:** Workforce development, Capacity, Health systems, Title V

## Abstract

*Objectives* A skilled workforce is essential to advancing maternal and child health (MCH) in a rapidly changing public health system. Little is known about the MCH workforce’s existing capacity to maximize opportunities afforded by ongoing change. We assessed MCH workforce capacity in three areas: Systems Integration, Evidence-Based Decision-Making, and Change Management/Adaptive Leadership. We then examined associations between workforce capacity and modifiable workforce development strategies/resources. *Methods* Data are from the Public Health Workforce Interests and Needs Survey (PH WINS). The present study was limited to employees working in MCH programs (weighted N = 3062). Workforce capacity was operationalized as self-reported awareness of public health trends and proficiency to perform related skills in the three areas. Survey-weighted generalized estimating equations were used to fit logistic regression models accounting for employee clustering within states. *Results* While awareness of public health trends was low, the majority of employees (> 70% in each area) reported proficiency to perform skills related to these trends. Capacity was lowest in Systems Integration. Employee engagement in academic partnerships and higher state contributions to MCH program budgets were the strategies/resources most consistently associated with higher capacity. Workplace support was the strongest correlate of capacity in Change Management/Adaptive Leadership. *Conclusions for Practice* Although employees lacked familiarity with specific public health trends, they were proficient in skills needed to engage in related work. Still, areas for improvement remain. Results provide a baseline against which future training efforts can be evaluated. Academic partnerships and MCH program funding may be useful to prioritize in the context of health transformation.

## Significance

Training today’s MCH workforce to maximize the opportunities afforded by a rapidly changing public health system is an urgent priority at local, state, and national levels. Little is known about the workforce’s existing capacity to translate new opportunities into improved health outcomes for women, children, and families. This study is the first to assess the national MCH workforce’s self-rated awareness of trends in public health practice, and their proficiency to perform related skills. Understanding existing capacity, and identifying effective workforce development strategies/resources to enhance capacity, is essential to the development of workforce training programs and the evaluation of their success.

## Introduction

The enactment of the Patient Protection and Affordable Care Act (ACA) in 2010, followed by transformation of the Title V Maternal and Child Health Services Block Grant in 2015, ushered in an era of change, innovation, and promise for the advancement of maternal and child health (MCH) in the United States. Today’s MCH workforce is faced with the exciting, yet challenging, feat of maximizing the opportunities afforded by these transformations. An aging workforce, budgetary restrictions, and limited formal leadership training further complicate the task of navigating new policies, procedures, and priorities (Grason et al. 2012). As such, training and development of the MCH workforce has become an urgent priority (Kavanagh 2015; Streeter 2015).

A comprehensive understanding of the MCH workforce’s existing capacity is critical for targeting training efforts, and evaluating their success. The Association of Maternal and Child Health Programs (AMCHP) routinely assesses workforce training, staffing, and development needs (Grason et al. 2012). In 2016, state Title V supervisors identified critical thinking, communication, and working with communities as the greatest training needs for their program staff (AMCHP 2016). Complementary and equally important insights may be gained from understanding how MCH workers rate their own capacity. In the context of health systems transformation, it may be particularly useful to examine how MCH workers rate their own knowledge of new trends in public health practice and their ability to perform related skills. Such information can support the development of relevant and responsive training efforts, and provide a baseline against which training impacts can be evaluated.

Effective development of workforce capacity requires numerous inputs. At a minimum, Public Health Accreditation Board (PHAB) standards require that training needs be assessed, opportunities for training and professional development offered, and a supportive work environment provided (Public Health Accreditation Board 2013). The availability of financial resources and the organizational structure of public health systems (Handler et al. 2001; Mays et al. 2006) may also affect workforce capacity. Increased public health spending (Mays et al. 2006) and health agency governance structure (e.g. centralized, decentralized, or shared government authority) (Hyde and Shortell 2012; Mays et al. 2006) have been associated with public health system performance. However, precisely how these strategies and resources are associated with MCH workforce capacity is unknown. While the above inputs likely work in tandem to affect capacity, some may be particularly important in the context of health systems transformation. Understanding which strategies and resources are most strongly associated with knowledge of new trends in public health practice and the ability to perform related skills, can guide decision-making regarding strategy prioritization and resource allocation.

**Fig. 1:**
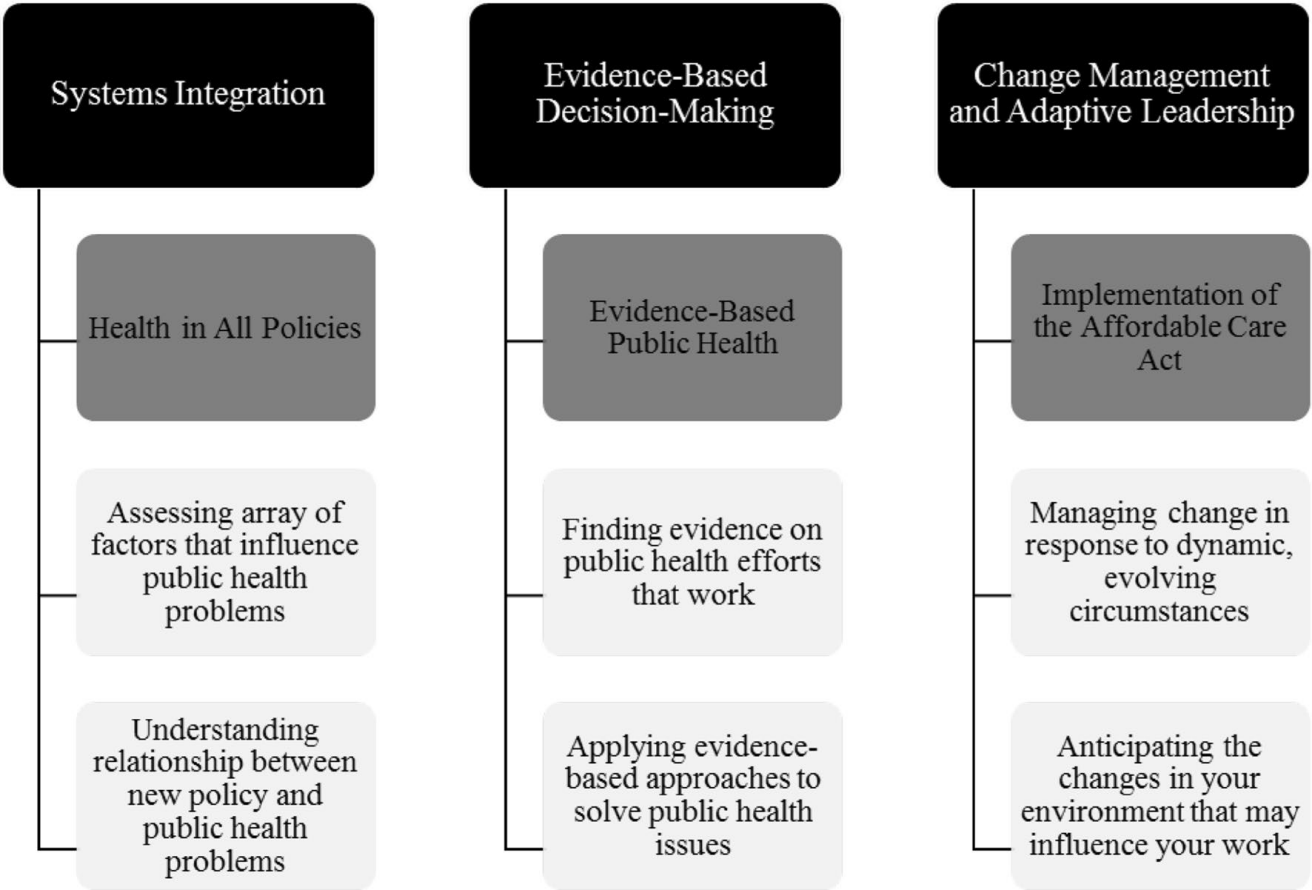
National MCH Workforce Development Center core training areas, related trends in public health practice, and associated job skills

The present study used data from the Public Health Workforce Interests and Needs Survey (PH WINS), the first nationally representative survey of state health agency (SHA) central office employees, to examine the capacity of the national MCH workforce to perform the complex work of health transformation. We operationalized capacity as employee awareness of broader trends in public health practice, and their proficiency to perform related skills, in three areas defined by the National MCH Workforce Development Center (the Center), a Maternal and Child Health Bureau (MCHB) funded cooperative agreement with UNC Chapel Hill, as critical to successful MCH leadership and practice (Fig. 1): Systems Integration, Evidence-Based Decision-Making, and Change Management/Adaptive Leadership (Margolis et al. 2017). The primary aim of this study was to describe the MCH workforce’s self-rated capacity in each of the Center’s three core areas, in order to highlight areas for improvement and provide a baseline against which future training efforts can be evaluated. The secondary aim was to conduct an exploratory analysis to identify modifiable workforce development resources and strategies associated with awareness and proficiency in each area. Specifically, we examined PHAB standards for workforce development (i.e. needs assessments, training and professional development opportunities, and workplace support), as well as the availability of financial resources (i.e. MCH program funding) and SHA governance structure.

## Methods

### Data Sources and Population

PH WINS was designed by the Association of State and Territorial Health Officials (ASTHO) and the de Beaumont Foundation to assess the knowledge, skills, and attitudes of the governmental public health workforce. The survey was fielded from September to December 2014 using web-based data collection methods. The primary sampling frame was designed to be representative of permanent SHA central office employees at the national level. Thirty-seven states chose to participate, and 10,246 responses were received from permanent SHA central office employees (46% response rate). Final sampling weights reflect the probability of selection, adjusted for differential nonresponse and post-stratified by Health and Human Services (HHS) geographic region, governance type, and size of population served. Additional detail on the PH WINS methodology is available elsewhere (Leider et al. 2015).

For the present study, the sample was restricted to employees who self-identified their program area as MCH, including WIC, and were not classified as administrative staff (n_employees_ = 704; weighted N_employees_ = 3062; n_states_ = 35).

We obtained additional data from the Title V Information System (TVIS) for use in exploratory analyses (detailed below). Unless otherwise indicated, variables were measured at the individual level.

### Measures

#### Workforce Capacity

As illustrated in Fig. 1, we operationalized workforce capacity in each of the three Center core areas (listed below) as awareness of broader trends in public health practice, and proficiency to perform related skills. The research team reached consensus on the categorization of trends and skills under each core area.

Systems Integration: awareness of Health in All Policies (HiAP) and proficiency to perform related skills: (1) assessing the broad array of factors that influence public health problems; (2) understanding the relationship between a new policy and public health problems.

Evidence-Based Decision-Making: awareness of Evidence-Based Public Health Practice (EBPH), and proficiency to perform related skills: (1) finding evidence on public health efforts that work; (2) applying evidence-based approaches to solve public health issues.

Change Management/Adaptive Leadership: awareness of Implementation of the ACA, and proficiency to perform related skills: (1) managing change in response to dynamic, evolving circumstances; (2) anticipating changes in the environment that may influence work.

For trends, participants were asked, “How much, if anything, have you heard about the following trends in public health?”; response options were grouped to create a binary variable indicating awareness (yes/no): “a lot” versus “nothing at all”, “not much”, or “a little.”

For skills, participants were asked to indicate their current skill level for each item; answer choices were grouped to create a binary variable indicating proficiency (yes/no): “proficient” or “expert” versus “unable to perform” or “beginner.”

#### Independent Variables for Exploratory Analysis

##### Needs Assessment

Participants were asked to rate their agreement with the statement, “My training needs are assessed.” Response options were grouped to create a binary variable (agree/disagree): “strongly agree” or “agree” versus “strongly disagree”, “disagree”, or “neither agree nor disagree.”

##### Training and Professional Development Opportunities

Training opportunities were operationalized as a 7-item continuous index. Participants were asked whether their health department engages in each of the following training opportunities (yes/no): requires continuing education; includes education and training objectives in performance reviews; allows use of working hours to participate in training; pays travel/registration fees for trainings; provides on-site training; has staff positions responsible for internal training; and provides recognition of achievement. The index was created by summing the number of “yes” responses (range 0–7).

We also assessed employee engagement in academic partnerships with the following yes or no question: “In the past year, have you worked with members of the academic community on public health practice issues?”.

##### Workplace Support

Workplace support was operationalized as a 6-item continuous index. Participants were asked to rate their agreement with six statements regarding support received from their supervisors: supervisors work well with employees of different backgrounds; supervisors in my work unit support employee development; my supervisor supports my need to balance work and family issues; my supervisor provides me with opportunities to demonstrate my leadership skills; my supervisor and I have a good working relationship, and my supervisor treats me with respect. Response options were grouped to create a binary variable for each item (agree/disagree): “strongly agree” or “agree” versus “strongly disagree”, “disagree”, or “neither agree nor disagree.” The index was created by summing the number of “agree” responses (range 0–6).

##### MCH Program Funding

State-level data from TVIS were used to create two continuous variables related to MCH program funding in FY 2015 (scaled so that a one-unit change represents a 10% increase): *percent of MCH budget from state MCH funding* (as states must match three out of every four federal dollars, higher percentages indicate that states overmatched federal funds to a greater degree), and *percent of MCH budget spent on Public Health Services and Systems (PHSS)* (e.g. activities related to core public health functions, including workforce development, versus direct and enabling health care services).

##### SHA Governance Structure

SHA governance structure was measured at the state-level. Participating states were categorized as: centralized or largely centralized; shared or largely shared; decentralized or largely decentralized; or mixed.

#### Control Variables

Exploratory analyses were adjusted for employee and state characteristics. Demographic and employment-related characteristics included: *education level* (bachelor’s degree or higher; no college degree), *role classification* (clinical/lab; public health science; social services), *years in public health practice* (continuous), *supervisory status* (supervisor, manager, or executive; non-supervisor or team leader), *gender* (male/female), and *race*/*ethnicity* (white; Black; Hispanic; other). Characteristics measured at the state level included: *paired HHS region* (New England and Atlantic; Mid-Atlantic and Great Lakes; South; Mountain and Midwest; West), and *size of population served* (small; medium; large).

### Analysis

Univariate and bivariate analyses were conducted to explore variable distributions and assess unadjusted significance of associations. Balanced Repeated Replication (BRR) variance methodology was used to estimate variance. Logistic regression models were run separately for each of the three public health trends and each of the six skills as outcomes using generalized estimating equations to account for the clustering of employees within states. Analyses were weighted to account for the complex survey design. For analyses with skill proficiency as the outcome, we excluded employees who indicated that their current position did not require them to perform the given skill. The unweighted analytic sample sizes for the six skills ranged from 569 to 606 (weighted N = 2455–2658). All multivariable models were assessed for collinearity. Missing data were handled through listwise deletion; no variables were missing greater than 2% of data. All analyses were conducted using SAS 9.4 (SAS Institute Inc., Cary, NC). The Chesapeake IRB deemed PH WINS exempt research.

## Results

### Characteristics of MCH Employees and SHAs

Table 1 presents characteristics of MCH employees and SHAs. The respondents were majority female (88%), non-Hispanic white (71%), and held a bachelor’s degree or higher (93%). The average tenure was 16 years; employees primarily held non-supervisory roles (58%), with the largest proportion working in public health sciences (e.g. program managers and associates) (60%) followed by clinical/lab positions (31%).

**Table 1:** Characteristics of maternal and child health workers and the state health agencies in which they work (weighted N = 3062)

	Weighted proportion	SE
Gender		
Female	87.8	1.42
Male	12.2	1.42
Race/ethnicity		
Black	13.6	2.68
White	71.1	1.99
Hispanic	4.6	0.89
Other	10.7	1.43
Education level		
Bachelor’s degree or higher	93.4	0.95
No college degree	6.6	0.95
Supervisory status		
Supervisor, manager, or executive	42.2	1.99
Non-supervisor or team leader	57.8	1.99
Role classification		
Clinical and lab	31.0	2.33
Public health science	60.1	3.18
Social services	8.9	1.39
Years in public health practice (*mean, SE*)	16.1	0.58
HHS region		
New England and Atlantic	16.7	1.76
Mid-Atlantic and Great Lakes	19.0	1.54
South	36.8	3.16
Mountain/Midwest	7.6	1.55
West	20.0	1.67
SHA governance structure		
Centralized/largely centralized	20.8	3.09
Shared/largely shared	14.0	2.51
Decentralized/largely decentralized	12.2	1.43
Mixed	53.1	2.90
Population served		
Small	16.9	2.05
Medium	29.7	2.84
Large	53.4	2.89

### Overall Trend Awareness and Skill Proficiency

**Fig. 2:**
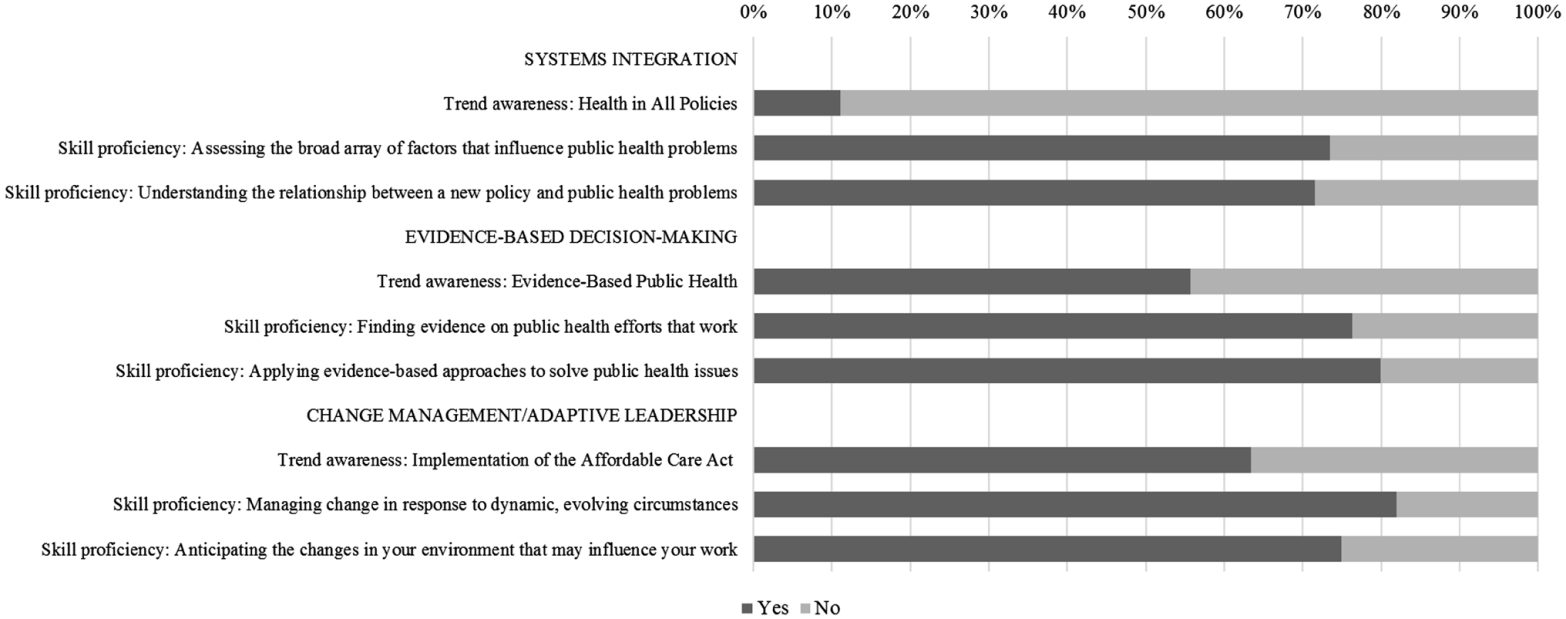
Weighted proportions estimating trend awareness and skill proficiency of Maternal and Child Health workers in State Health Agency central offices (weighted N = 2632–3057). Trend awareness classified as *Yes* if employee reported having heard “a lot” and *No* if employee reported “nothing at all”, “not much”, or “a little”; Skill proficiency classified as *Yes* if employee reported being “proficient” or “expert” and *No* if employee reported being “unable to perform” or “beginner”. N varies by trend and skill as employees who indicated that their current position did not require them to perform a given skill were excluded from analyses. N does not equal full sample size due to missing data

Within each of the three core areas, the proportion of MCH employees reporting skill proficiency was substantively higher than the proportion reporting awareness of the related trend (Fig. 2). Within the Systems Integration core, only 11% reported awareness of HiAP, yet 74% reported proficiency to assess the broad array of factors that influence public health problems, and 72% reported proficiency to understand the relationship between a new policy and public health problems. Similarly, within the Evidence-Based Decision-Making core, a little over half (56%) reported awareness of EBPH, while 76% reported proficiency to find evidence on public health efforts that work, and 80% reported proficiency to apply evidence-based approaches to solve public health issues. In the Change Management/Adaptive Leadership core, 63% of MCH employees reported awareness of Implementation of the ACA, while 82% reported proficiency to manage change in response to dynamic, evolving circumstances, and 75% reported proficiency to anticipate changes in the environment that may influence work.

### Workforce Development Strategies and Resources Associated with Trend Awareness and Skill Proficiency

#### Systems Integration

In the Systems Integration core area (Table 2), MCH employees who engaged in academic partnerships had higher odds of awareness of HiAP (aOR 2.46; 95% CI 1.13, 5.35) and higher odds of proficiency to assess the broad array of factors that influence public health problems (aOR 2.37; 95% CI 1.43, 3.94) and understand the relationship between a new policy and public health programs (aOR 2.18; 95% CI 1.51, 3.13). Each additional workplace training opportunity was associated with higher odds of proficiency to understand the relationship between a new policy and public health programs (aOR 1.23; 95% CI 1.04, 1.44).

**Table 2:** Workforce development inputs associated with trend awareness and skill proficiency in *Systems Integration*

	Awareness of health in all policies^a^ (weighted n = 2810)^c,d^			Skill proficiency for assessing the broad array of factors that influence public health problems^b^ (weighted n = 2532)^c,d^			Skill proficiency for understanding the relationship between a new policy and public health problems^b^ (weighted n = 2515)^c,d^		
	aOR	CI		aOR	CI		aOR	CI	
Training needs assessed	1.62	0.78	3.36	0.72	0.44	1.19	1.23	0.78	1.94
Training opportunities (continuous)	1.13	0.95	1.34	1.05	0.92	1.20	1.23*	1.04	1.44
Academic partnerships (ref = no academic partnerships)	2.46*	1.13	5.35	2.37**	1.43	3.94	2.18***	1.51	3.13
Workplace support (continuous)	1.04	0.88	1.24	0.99	0.86	1.13	0.94	0.82	1.06
SHA governance structure (ref = centralized/largely centralized)									
Shared/largely shared	1.06	0.31	3.59	3.00**	1.40	6.43	2.13	0.57	7.97
Decentralized/largely decentralized	0.40	0.09	1.75	2.06*	1.18	3.58	0.98	0.48	1.99
Mixed	3.29	0.96	11.34	2.70**	1.34	5.43	1.45	0.46	4.55
% MCH budget from state funding (continuous)	1.24**	1.08	1.44	0.92	0.78	1.08	1.13	0.96	1.33
% MCH budget for Public Health Services and Systems (continuous)	1.12*	1.00	1.25	0.86**	0.79	0.95	0.88**	0.79	0.96

*p < 0.05; **p < 0.01; ***p < 0.0001

^a^Nothing/not much/a little versus a lot

^b^Unable to perform/beginner versus proficient/expert

^c^N varies by trend and skill as employees who indicated that their current position did not require them to perform a given skill were excluded from analyses

^d^N does not equal full sample size due to listwise deletion of missing data

With regard to MCH program funding, each additional 10% of the state MCH budget that came from state funding was associated with higher odds of awareness of HiAP (aOR 1.24; 95% CI 1.08, 1.44), and each additional 10% of the state MCH budget allocated to PHSS (vs. direct or enabling services) was associated with higher odds of awareness of HiAP (aOR 1.12; 95% CI 1.00, 1.25). In contrast, each additional 10% of the budget allocated to PHSS was associated with lower odds of reported proficiency in both skills. Employees who worked in states with shared, decentralized, or mixed SHA governance structures had higher odds of reporting proficiency to assess the broad array of factors that influence public health problems (aOR 3.00; 95% CI 1.40, 6.43; aOR 2.06; 95% CI 1.18, 3.58, aOR 2.70; 95% CI 1.34, 5.43, respectively), compared to employees working in centralized SHAs.

#### Evidence-Based Decision-Making

Similar workforce development strategies and resources were salient in the Evidence-Based Decision-Making core area (Table 3). MCH employees who engaged in academic partnerships had higher odds of awareness of EBPH (aOR 3.22; 95% CI 2.03, 5.11) than those not engaged in academic partnerships, as well as higher odds of proficiency in finding evidence on public health efforts that work (aOR 2.03; 95% CI 1.30, 3.17), and applying evidence-based approaches to solve public health issues (aOR 4.05; 95% CI 2.59, 6.33). For each additional employee training opportunity, MCH employees had higher odds of awareness of EBPH (aOR 1.14; 95% CI 1.04, 1.25).

**Table 3:** Workforce development inputs associated with trend awareness and skill proficiency in *Evidence-Based Decision-Making*

	Awareness of evidence-based public health^a^ (weighted n = 2796^c,d^)			Skill proficiency for finding evidence on public health efforts that work^b^ (weighted n = 2455^c,d^)			Skill proficiency for applying evidence-based approaches to solve public health issues^b^ (weighted n = 2540^c,d^)		
	aOR	CI		aOR	CI		aOR	CI	
Training needs assessed	1.09	0.64	1.86	1.20	0.73	1.96	1.56	0.91	2.64
Training opportunities (continuous)	1.14**	1.04	1.25	1.10	0.89	1.36	1.10	0.97	1.25
Academic partnerships (ref = no academic partnerships)	3.22***	2.03	5.11	2.03**	1.30	3.17	4.05***	2.59	6.33
Workplace support (continuous)	0.95	0.81	1.10	1.02	0.90	1.17	0.91	0.78	1.06
SHA governance structure (ref = centralized/largely centralized)									
Shared/largely shared	1.79	0.88	3.61	1.22	0.52	2.86	0.81	0.30	2.20
Decentralized/largely decentralized	1.77	0.93	3.37	1.36	0.72	2.57	1.50	0.57	3.93
Mixed	1.89*	1.02	3.48	1.73	0.82	3.64	1.10	0.41	2.96
% MCH budget from state funding (continuous)	1.14**	1.06	1.22	1.17**	1.06	1.30	1.18**	1.08	1.30
% MCH budget for Public Health Services and Systems (continuous)	0.89**	0.84	0.95	0.94	0.85	1.04	0.89*	0.80	0.99

*p < 0.05; **p < 0.01; ***p < 0.0001

^a^Nothing/not much/a little versus a lot

^b^Unable to perform/beginner versus proficient/expert

^c^N varies by trend and skill as employees who indicated that their current position did not require them to perform a given skill were excluded from analyses

^d^N does not equal full sample size due to listwise deletion of missing data

Regarding MCH program funding, each additional 10% of the state MCH budget that came from state funding was associated with higher odds of awareness of EBPH (aOR 1.14; 95% CI 1.06, 1.22) and higher odds of proficiency in both related skills. In contrast, each additional 10% of the budget allocated to PHSS was associated with lower odds of awareness of EBPH (aOR 0.89; 95% CI 0.84, 0.95) and proficiency in applying evidence-based approaches to solve public health issues (aOR 0.89; 95% CI 0.80–0.99). Employees in SHAs with mixed governance structures had higher odds of awareness of EBPH (aOR 1.89; 95% CI 1.02, 3.48).

#### Change Management/Adaptive Leadership

In the Change Management/Adaptive Leadership domain (Table 4), MCH employees who engaged in academic partnerships had higher odds of awareness of ACA implementation (aOR 2.54; 95% CI 1.89, 3.42). Unlike the preceding core areas, academic partnerships were not associated with proficiency in either skill. Each additional training opportunity was associated with higher odds of awareness of ACA implementation (aOR 1.23; 95% CI 1.08, 1.41). Change Management/Adaptive Leadership was the only domain in which supervisory support was associated with skill proficiency: each additional support item endorsed was associated with higher odds of proficiency in anticipating changes in the environment that may influence work (aOR 1.24; 95% CI 1.09, 1.41).

**Table 4:** Workforce development inputs associated with awareness and skill proficiency in *Change Management and Adaptive Leadership*

	Implementation of the Affordable Care Act^a^ (weighted n = 2811^c,d^)			Skill proficiency for Managing change in response to dynamic, evolving circumstances^b^ (weighted n = 2658^c,d^)			Skill proficiency for Anticipating the changes in your environment that may influence your work^b^ (weighted n = 2553^c,d^)		
	aOR	CI		aOR	CI		aOR	CI	
Training needs assessed	0.77	0.45	1.30	1.63	0.86	3.09	0.78	0.48	1.26
Training opportunities (continuous)	1.23**	1.08	1.41	0.99	0.84	1.16	1.00	0.86	1.17
Academic partnerships (ref = no academic partnerships)	2.54***	1.89	3.42	1.36	0.78	2.36	1.17	0.78	1.76
Workplace support (continuous)	1.05	0.96	1.16	1.01	0.87	1.18	1.24**	1.09	1.41
SHA governance structure (ref = centralized/largely centralized)									
Shared/largely shared	0.97	0.47	1.97	1.66	0.66	4.17	1.25	0.40	3.88
Decentralized/largely decentralized	0.61	0.34	1.09	0.70	0.32	1.56	0.98	0.46	2.05
Mixed	1.00	0.45	2.21	0.84	0.28	2.49	0.98	0.30	3.23
% MCH budget from state funding (continuous)	0.95	0.87	1.04	1.24**	1.07	1.44	1.21*	1.04	1.41
% MCH budget for Public Health Services and Systems (continuous)	0.87**	0.80	0.95	1.05	0.94	1.17	0.88**	0.80	0.96

*p < 0.05, **p < 0.01; ***p < 0.0001

^a^Nothing/not much/a little versus a lot

^b^Unable to perform/beginner versus proficient/expert

^c^N varies by trend and skill as employees who indicated that their current position did not require them to perform a given skill were excluded from analyses

^d^N does not equal full sample size due to listwise deletion of missing data

Similar to the preceding core areas, each additional 10% of the state MCH budget that came from state funding was associated with higher odds of proficiency in managing change in response to dynamic, evolving circumstances (aOR 1.24; 95% CI 1.07, 1.44) and anticipating changes in the environment that may influence work (aOR 1.21; 95% CI 1.04, 1.41). Each additional 10% of the state MCH budget allocated to PHSS was associated with lower odds of awareness of ACA implementation (aOR 0.87; 95% CI 0.80, 0.95) and proficiency to anticipate changes in the environment that may influence work (aOR 0.88; 95% CI 0.80, 0.96).

## Discussion

Overall, our findings suggest that SHA MCH employees possess the skills needed to engage in the complex work of health transformation: despite lacking familiarity with trends in public health practice by name, the majority (over 70% in each area) reported proficiency to perform related skills. Still, areas for improvement remain. Awareness and skill proficiency were lowest in the Systems Integration core, and a sizable minority of MCH employees were not familiar with Evidence-Based Public Health or Implementation of the ACA. In exploratory analyses, engagement in academic partnerships and MCH program funding were the workforce development inputs most consistently associated with capacity across core areas. Findings also suggest that training may require tailoring by skill type. Although engaging in academic partnerships was the strongest correlate of workforce capacity in the Systems Integration and Evidence-Based Decision-Making core areas, supervisory support was the strongest correlate of skill proficiency in the Change Management/Adaptive Leadership core.

Engagement in academic partnerships emerged as a strong correlate of awareness and skill proficiency across core areas. The centrality of academic-practitioner partnerships to successful public health programming is well recognized, yet many challenges remain to cultivating a broader acceptance of practice-based research and evidence (Ammerman et al. 2014; Dwelle et al. 2015). The Center and other MCHB programs, such as the Centers of Excellence in MCH Education, Science, and Practice, foreground academic-practice partnerships as a critical strategy for workforce training and development and are making a concerted effort to narrow the gap between research and practice; the current findings provide additional support for the importance of this approach.

Training opportunities were also relevant to either awareness or skill proficiency in each core area. Ensuring that MCH employees benefit from these opportunities requires addressing the training barriers AMCHP identified in its most recent assessment—cost of continuing education, travel restrictions, and difficulty taking time from work (AMCHP 2016). The finding that supervisory support was the strongest correlate of skill proficiency in the Change Management/Adaptive Leadership domain further highlights the importance of the work environment. Perceived supervisory support is a well-established predictor of employee job satisfaction, commitment, and performance (Rhoades and Eisenberger 2002). In the context of health transformation, it may be particularly salient to cultivating change management and leadership skills.

In examining financial resources, employees working in MCH programs where state funding comprised a higher percentage of the MCH budget had higher odds of awareness and skill proficiency across core areas. Our findings suggest that the MCH workforce may benefit from states’ ability and decision to overmatch federal funds and align with previous research demonstrating population-level health benefits of increased public health expenditures (Mays and Smith 2011; Singh 2014). Yet, the degree to which states are able to overmatch funds likely depends on the social, political, and economic climates of the state (Handler et al. 2001). Further, whether benefits accrue directly through the availability of more resources for workforce development, or indirectly via a more complex set of mechanisms that affect capacity requires further study.

In contrast, states that allocated a greater percentage of their budget toward PHSS generally had lower odds of awareness and skill proficiency. Given the recent realignment of the MCH Pyramid of Health Services to prioritize PHSS activities, including workforce development, needs assessment, quality improvement, and policy development, we expected to find the opposite association. A possible explanation for our finding is that states that allocate more funds to PHSS do so *because* they are seeking to address lower capacity or performance. Further research on the relationship between workforce capacity and states’ budget allocations for Title V and PHSS is needed.

Mixed governance structures were associated with skill proficiency in the Systems Integration core, and awareness in the Evidence-Based Decision-Making core, suggesting that there may be benefits to more collaborative governance structures. Mays et al. (2006) found that local health departments with mixed or shared governance structures performed better on essential public health services compared to centralized or decentralized systems, and suggested that the results may be attributable to the combined effects of state-level infrastructure and local flexibility. However, existing evidence on the effects of governance structure on performance remains mixed (Hyde and Shortell 2012).

Our findings may help inform how our field approaches the provision of training and technical assistance (TA). Although SHA MCH employees possessed the skills needed to perform their work, there may be added value in the ability to recognize and think critically about trends in public health practice. Such recognition may help workers connect to resources and training opportunities, or make them more conversant with peers and partners. Organizations responsible for training and TA cannot assume that workers are familiar with a topic because it has been prioritized and widely discussed at the national level. Further, for training efforts to be effective over the long-term, they must be accompanied by efforts to retain skilled workers, and cultivate enduring leadership across MCH programs.

## Limitations

This study is subject to several limitations. Capacity was self-reported and alternative measurement approaches may produce different results. The trends and skills used to represent capacity in each Center core area are not intended to provide a comprehensive assessment of all relevant indicators of capacity. Our analyses were exploratory and it is possible that the independent variables in our models relate to one another and the outcome in more complex ways (i.e. as mediators or effect modifiers). Future research can advance our analysis by examining theoretically driven associations between select predictors and outcomes. Finally, we included Title V and WIC employees, despite differences in their roles, responsibilities, and funding sources, as many state MCH programs incorporate both and do not differentiate between the employees for training purposes.

## Conclusion

The ongoing transformation of U.S. health systems has created numerous opportunities for the advancement of MCH. Such changes are not without challenge, requiring workforce flexibility and dynamic approaches to training and development. Our findings suggest that the MCH workforce is well positioned to maximize new opportunities, but that areas for improvement remain. Future training efforts may benefit from targeting areas in which awareness and proficiency are low, such as Systems Integration, and tailoring approaches to the skillset in question. How workforce development strategies and resources affect capacity in the context of health transformation requires further study; their identification provides the opportunity to replicate factors that enhance workforce capacity and address those that impede development.
